# A conserved domain of *Drosophila* RNA-binding protein Pumilio interacts with multiple CCR4–NOT deadenylase complex subunits to repress target mRNAs

**DOI:** 10.1016/j.jbc.2022.102270

**Published:** 2022-07-16

**Authors:** Rebecca J. Haugen, René M. Arvola, Robert P. Connacher, Richard T. Roden, Aaron C. Goldstrohm

**Affiliations:** 1 Department of Biochemistry, Molecular Biology, and Biophysics, University of Minnesota, Minneapolis, Minnesota, USA; 2 Department of Biological Chemistry, University of Michigan, Ann Arbor, Michigan, USA

**Keywords:** Pumilio, CCR4–NOT, deadenylase, mRNA regulation, cDNA, complementary DNA, CR, conserved region, DDO, double dropout, EGFP, enhanced GFP, HA, hemagglutinin, HRP, horseradish peroxidase, PRE, Pumilio response element, qPCR, quantitative PCR, RBD, RNA-binding domain, RD, repression domain, SDM, Schneider’s Drosophila medium, TBST, Tris-buffered saline with Tween-20

## Abstract

Pumilio is a sequence-specific RNA-binding protein that controls development, stem cell fate, and neurological functions in *Drosophila*. Pumilio represses protein expression by destabilizing target mRNAs in a manner dependent on the CCR4–NOT deadenylase complex. Three unique repression domains in the N-terminal region of Pumilio were previously shown to recruit CCR4–NOT, but how they do so was not well understood. In this study, we identified the motifs that are necessary and sufficient for the activity of the third repression domain of Pumilio, designated RD3, which is present in all isoforms and has conserved regulatory function. We identified multiple conserved regions of RD3 that are important for repression activity in cell-based reporter gene assays. Using yeast two-hybrid assays, we show that RD3 contacts specific regions of the Not1, Not2, and Not3 subunits of the CCR4–NOT complex. Our results indicate that RD3 makes multivalent interactions with CCR4–NOT mediated by conserved short linear interaction motifs. Specifically, two phenylalanine residues in RD3 make crucial contacts with Not1 that are essential for its repression activity. Using reporter gene assays, we also identify three new target mRNAs that are repressed by Pumilio and show that RD3 contributes to their regulation. Together, these results provide important insights into the mechanism by which Pumilio recruits CCR4–NOT to regulate the expression of target mRNAs.

RNA-binding proteins control the fate of mRNAs (1, 2). The sequence-specific RNA-binding protein Pumilio from *Drosophila melanogaster* is a prime example (3). Pumilio selectively binds with high affinity to hundreds of target mRNAs by recognizing Pumilio response elements (PREs; 5′-UGUANAUA) that are predominantly located in 3′UTR (3, 4). PRE RNA is recognized by the C-terminal RNA-binding domain (RBD) of Pumilio, which is composed of eight alpha-helical repeats and is the defining feature of the eukaryotic family of Pumilio and Fem3-binding factor (PUF) proteins (Fig. 1) (3, 5, 6, 7).

**Figure 1:**
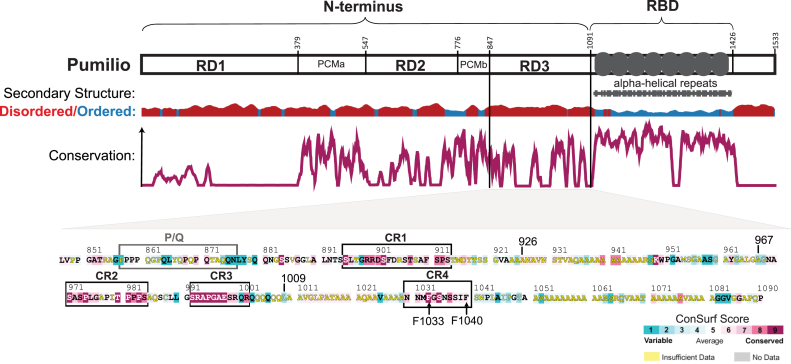
**Domain architecture and sequence conservation of *Drosophila* Pumilio.** Diagram of *Drosophila* Pumilio protein showing functional domains and secondary structure. The predicted disordered *versus* ordered regions were computed by JRONN, with the height of the plot (*y*-axis) indicating the probability of disorder (order) or order (*blue*) per amino acid residue (*x*-axis) (47, 48). Functional domains include three repression domains (RD1–RD3), Pumilio conserved motifs (PCMa and PCMb), and the Pum-HD/PUF repeats (R1–R8) of the RNA-binding domain (RBD) (9, 16). Conservation of Pumilio proteins is plotted as the relative sequence conservation (*y*-axis) *versus* amino acid residue position (*x*-axis) based on comparison of 82 Pumilio protein orthologs from species spanning insects, fish, reptiles, birds, marsupials, mammals, primates, and humans, generated using Clustal Omega, Consurf, and Emboss Plotcon (www.bioinformatics.nl/cgi-bin/emboss/plotcon) (16, 49). The amino acid sequence of RD3 region is shown at the *bottom*, with conserved regions, CR1–CR4, and the P/Q region indicated by *boxed* sequences. Conservation of the amino acids is indicated according to the ConSurf Score scale shown in the *lower right* (49).

Pumilio is a repressor that reduces protein expression from its target mRNAs by accelerating their degradation (3, 8, 9). The CCR4–NOT deadenylase complex is a crucial component of the Pumilio-mediated repression mechanism (8). CCR4–NOT is responsible for initiating mRNA degradation pathways, catalyzed by two deadenylase subunits, Pop2 and Ccr4 (10, 11). CCR4–NOT has emerged as a pivotal nexus for regulation of mRNA fate (12, 13). Multiple components of the eight subunit CCR4–NOT complex are necessary for Pumilio repression activity in *Drosophila* cells, including Pop2 and the structural backbone of the complex, Not1. The Not2 and Not3 subunits also contribute to Pumilio repression (8, 14). While biochemical analyses showed that Pumilio associates with CCR4–NOT (8, 14, 15), the molecular basis of these functional requirements remains to be elucidated.

Multiple regions of Pumilio contribute to its ability to repress target mRNAs. The RBD is well characterized structurally and functionally, revealing that—in addition to PRE recognition—it promotes CCR4–NOT mediated deadenylation and antagonizes the activity of poly(A)-binding protein, PABP (5, 7, 14). However, the RBD makes a minor contribution to Pumilio repression activity (9, 14). The major repression activity of Pumilio is conferred by its large N-terminal region (Fig. 1), which is unique to Pumilio orthologs spanning insects to vertebrates (3, 9). It is characterized by conserved regions (CRs) that are interspersed by more rapidly evolving sequences (Fig. 1). The N-terminal region lacks structural homology and is predicted to be intrinsically disordered (Fig. 1). Previous analyses mapped three repression domains (Fig. 1. RD1–3) within the N-terminal region, each of which can reduce protein expression and cause mRNA decay when directed to a transcript (9). They appear to function redundantly and can be individually deleted without compromising Pumilio’s repressive activity (9). The RDs do not share sequence homology (9), but they each interact with and require the CCR4–NOT complex for repression activity (8). We seek to determine how Pum RDs contact CCR4–NOT, in order to fully understand their regulatory mechanisms and function *in vivo*.

In this study, we dissect the molecular mechanism of repression by Pumilio, focusing on the third RD, RD3, for several reasons. First, RD3 is the best conserved of the three N-terminal RDs (Fig. 1) (9, 16). Second, RD3 is the only RD present in all eight annotated Pumilio isoforms in *Drosophila*. Third, the repressive function of RD3 is conserved by the human orthologs, PUM1 and PUM2 (9, 17). We previously reported that RD3 associates with the CCR4–NOT complex; however, the precise contacts were unknown (8). Here, we identify the motifs of RD3 that are necessary and sufficient for its activity. We show that RD3 interacts with three of the eight subunits of the CCR4–NOT complex. We also identify the determinants necessary for each interaction, including conserved motifs and amino acid residues that are necessary for repression activity and specific protein interactions. Collectively, the results support a model wherein RD3 contains short linear interaction motifs that contact CCR4–NOT and mediate its recruitment to target mRNAs.

## Results

### Identification of functional determinants of Pumilio RD3

RD3 of *Drosophila* Pumilio encompasses amino acids 847 to 1090 and shares 23% amino acid identity with human Pumilio orthologs (9). RD3 does not share homology to known structural domains and is predicted to be predominantly disordered (Fig. 1). Through sequence comparisons of 82 metazoan Pumilio orthologs, we identified four CRs, designated CR1–4 (Fig. 1) (16). RD3 is also interspersed with low complexity sequences enriched with alanine, glutamine, or proline. One proline and glutamine-rich region (P/Q) has moderate conservation, whereas other low complexity sequences are not conserved (Fig. 1). Based on these characteristics, we hypothesized that the CRs of RD3 may function as short linear interact motifs (18, 19) that could bind to protein cofactors necessary for repression.

**Figure 2:**
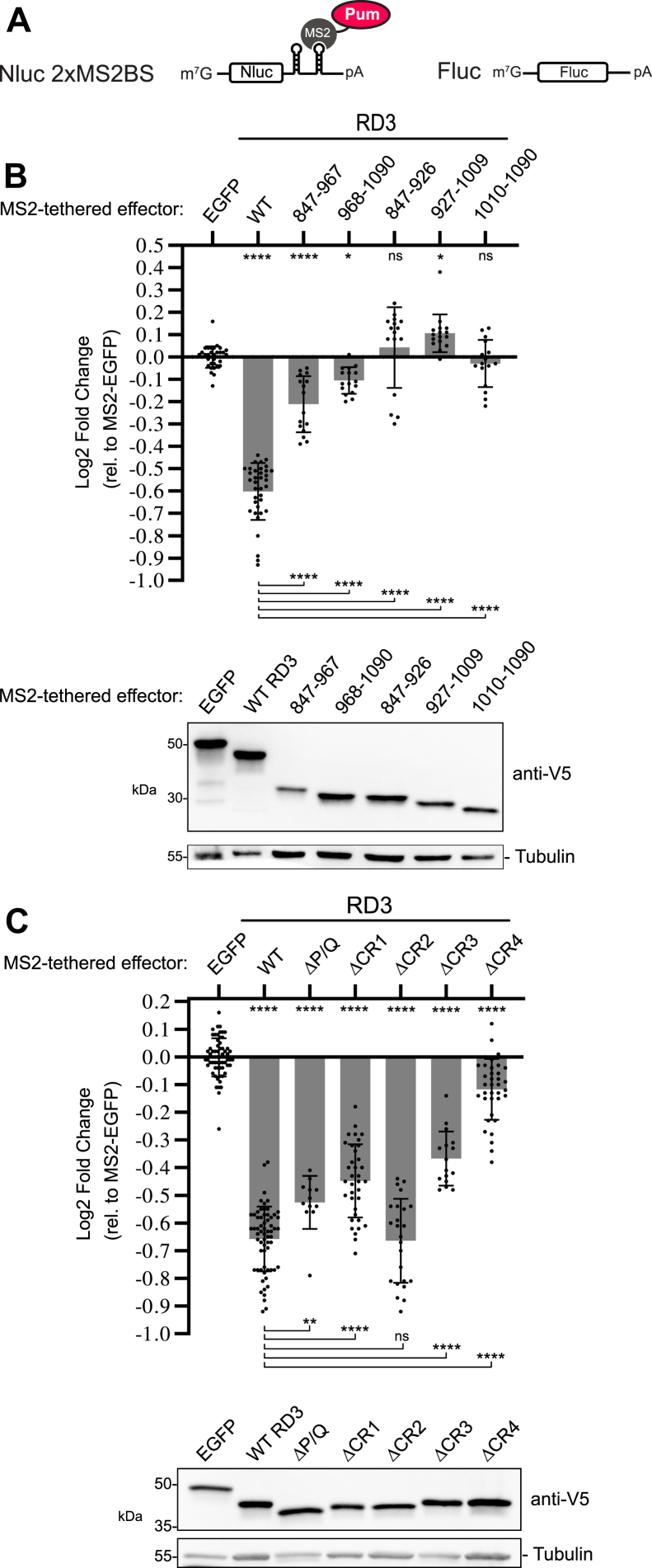
**Identification of functional determinants of Pumilio repression domain 3.***A*, diagrams of the Nano-luciferase reporter gene (Nluc 2×MS2BS) and the internal control firefly luciferase gene (Fluc) used in tethered function assays. The Pumilio test proteins or negative control were fused to the RNA-binding domain derived from the MS2 phage coat protein and directed to the Nluc reporter’s 3′UTR that contains two MS2-binding sites. *B*, mean and replicate log2 fold change values in normalized tethered function reporter activity, measured relative to negative-control MS2-EGFP, are plotted for each Pumilio RD3 test protein, indicated at the *top*. The amino acid residues contained in each RD3 test construct are labeled corresponding to Figure 1. N ≥ 16, ±StDev. Significance is indicated *above* the axis relative to MS2-EGFP, whereas those indicated *below* the axis were calculated relative to wildtype MS2-RD3. For significance calling, *p* < 0.05 = ∗, *p* < 0.01 = ∗∗, *p* < 0.001 = ∗∗∗, and *p* < 0.0001 = ∗∗∗∗ based on ordinary one-way ANOVA and Tukey's test for multiple comparisons. All data, including the plotted replicate and mean values and statistics, are reported in Table S1. Western blot confirming the expression of each test protein is shown at the *bottom*. MS2 fusion proteins were detected *via* their C-terminal V5 epitope tag. Tubulin served as a loading control. *C*, mean and replicate log2 fold change values for each RD3 test protein, indicated at the *top*, including deletions of the P/Q, CR1–4 regions, as indicated in Figure 1. N ≥ 12, ±StDev. At the *bottom*, Western blot confirmed the expression of each V5-tagged test protein. EGFP, enhanced GFP.

To begin to functionally dissect RD3, we used an established tethered function assay that specifically measures the repressive activity of RD3 (8, 9, 20). When fused to the RBD of MS2 phage coat protein, RD3 represses a Nano-luciferase reporter mRNA that has high-affinity MS2-binding sites in its 3′UTR (Fig. 2*A*, Nluc 2xMS2BS). Repression by RD3 was previously shown to be manifested in reduced Nano-luciferase reporter protein and mRNA levels (8, 9, 20). Cotransfected firefly luciferase (Fluc) gene served as a control for normalization of transfection efficiency (21). We note that each test was performed in a minimum of three independent experiments, each with at least four biological replicates unless otherwise noted. In all experiments, repression of the reporter was measured relative to the negative control, MS2-tethered enhanced GFP (MS2-EGFP) (8). Furthermore, all experiments included the positive control, MS2-tethered wildtype Pumilio RD3. The data (including individual replicate data and mean values) and statistics for each graph are reported in Table S1. Statistical significance of the observations was assessed by ordinary one-way ANOVA, unless otherwise noted in the figure legends and data tables.

We first compared wildtype RD3 activity to several truncated versions (Fig. 2*B*). The two halves of RD3 displayed substantially reduced activity relative to wildtype (Fig. 2*B*; amino acids 847–967 and 968–1090), whereas the thirds of RD3 were inactive (Fig. 2*B*; amino acids 847–926, 927–1009, and 1010–1090). These results indicate that RD3 is a functional unit that contains features that ostensibly cannot be separated.

To identify the features of RD3 necessary for repression, we focused on the CR1–4 (Fig. 1). Because proline-rich motifs can serve as short linear interact motifs (22), we also examined the P/Q-rich region. Deletion of CR4 caused a substantial loss of RD3 activity (Fig. 2*C*). We also observed that deletions of CR1, CR3, and P/Q reduced RD3 activity to a lesser degree. In contrast, deletion of CR2 had no effect. These results indicate that multiple CRs contribute to the repressive activity of RD3.

**Figure 3:**
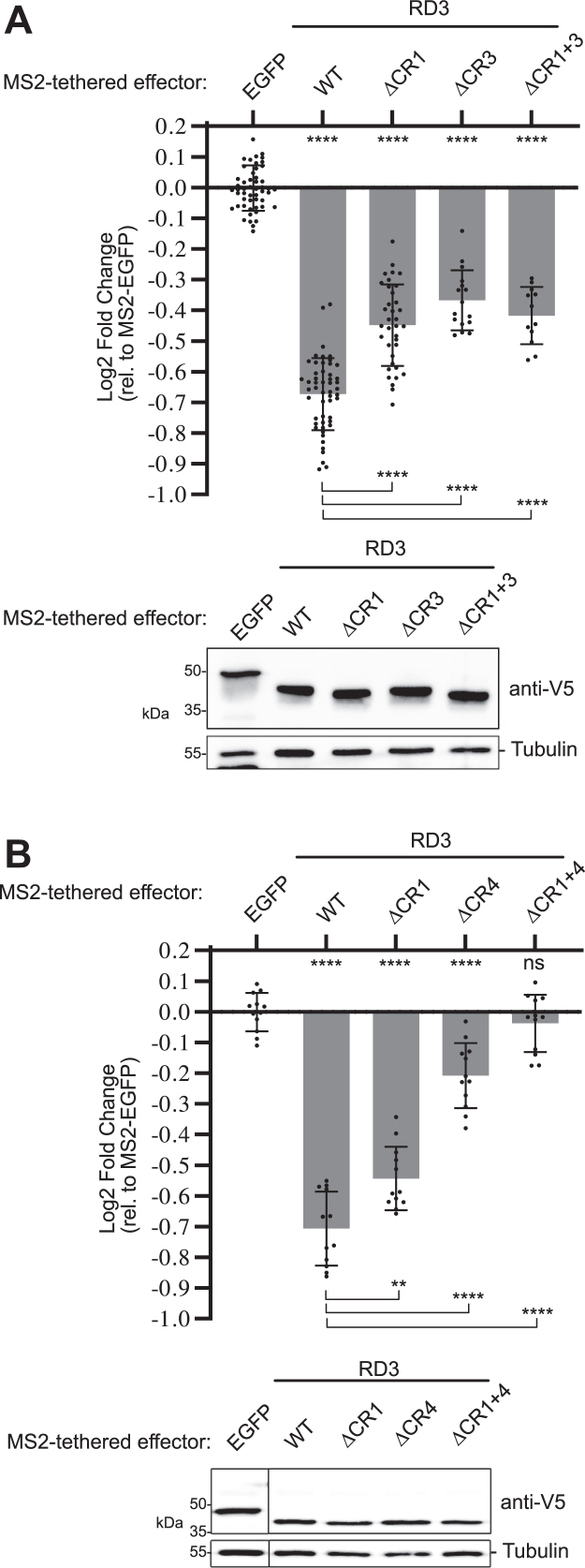
**Conserved regions CR1 and CR4 are necessary for Pumilio RD3 repression activity.***A*, mean and replicate log2 fold change values of wildtype RD3 or versions with deletions of CR1, CR3, or both, as measured in tethered function assays. N ≥ 12, ±StDev. Significance is indicated *above* the axis relative to MS2-EGFP, whereas *below* the axis relative to wildtype MS2-RD3. For significance calling, *p* < 0.05 = ∗, *p* < 0.01 = ∗∗, *p* < 0.001 = ∗∗∗, and *p* < 0.0001 = ∗∗∗∗ based on ordinary one-way ANOVA and Tukey's test for multiple comparisons. All data, including the plotted replicate and mean values and statistics, are reported in Table S1. Western blot confirming the expression of each V5-tagged test protein is shown at the *bottom*. Tubulin served as a loading control. *B*, mean and replicate log2 fold change values of wildtype RD3 or version with deletions of CR1, CR4, or both. N = 12, ±StDev. Western blot confirming the expression of each V5-tagged test protein is shown at the *bottom*.

We then tested the effect of combined deletions of the important RD3 CRs. Deletion of both CR1 and CR3 reduced repression relative to wildtype RD3 but not more than their individual deletions (Fig. 3*A*). In contrast, deletion of both CR1 and CR4 inactivated RD3 (Fig. 3*B*). This observation suggests that CR1 and CR4 may function together to cause repression.

**Figure 4:**
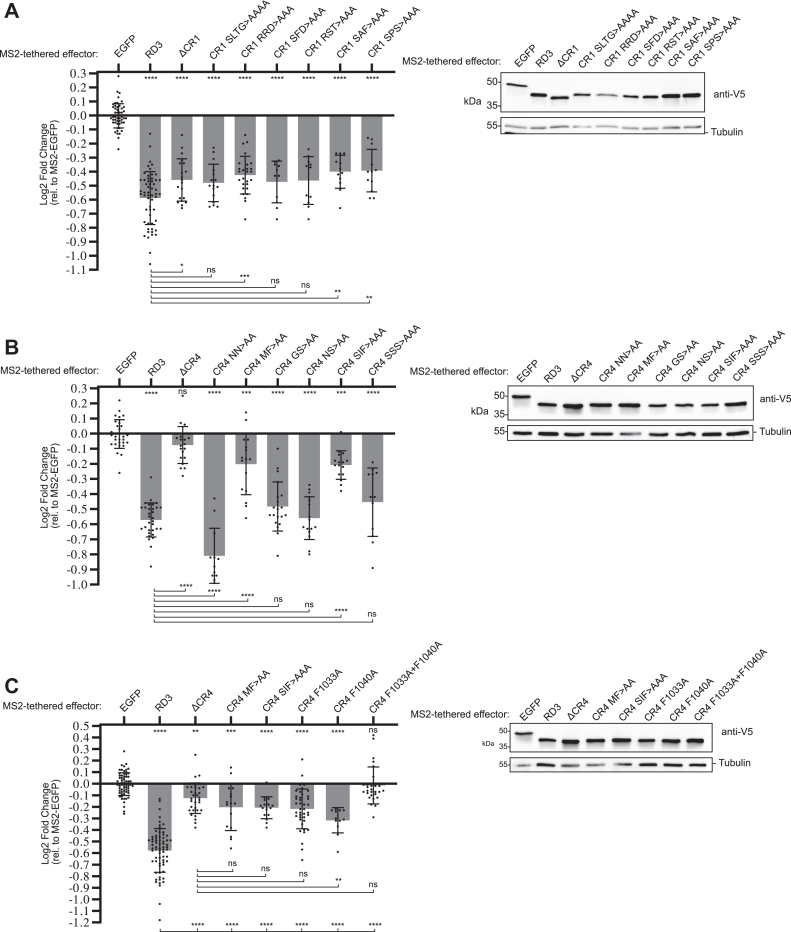
**Identification of functional amino acid residues in CR1 and CR4 of Pumilio RD3.***A*, mean and replicate log2 fold change values of wildtype or CR1 mutant versions of MS2-RD3 as measured in tethered function assays. N ≥ 11, ±StDev. Significance is indicated *above* the axis relative to MS2-EGFP, whereas *below* the axis relative to wildtype MS2-RD3. For significance calling, *p* < 0.05 = ∗, *p* < 0.01 = ∗∗, *p* < 0.001 = ∗∗∗, AND *p* < 0.0001 = ∗∗∗∗ based on ordinary one-way ANOVA and Tukey's test for multiple comparisons. All data, including the plotted replicate and mean values and statistics, are reported in Table S1. Western blot confirmed the expression of each V5-tagged test protein, as shown at the *bottom*. Tubulin served as a loading control. *B* and *C*, mean and replicate log2 fold change values of wildtype and CR4 mutant version of MS2-RD3. N ≥ 12, ±StDev. In *C*, additional significance tests were performed relative to the CR4 deletion, as indicated *below* the axis. EGFP, enhanced GFP.

To identify crucial amino acids, we performed alanine scanning mutagenesis of CR1 and CR4. Multiple mutations in CR1 reduced its repression activity to a similar degree as its deletion (Fig. 4*A*). For CR4, alanine substitutions of M1032 and F1033, or S1038, I1039, and F1040, significantly reduced RD3 activity (Fig. 4*B*). Most dramatically, combined F1033A and F1040A mutations inactivated RD3 (Fig. 4*C*). The other CR4 mutations had little or no effect, except for the alanine substitutions of N1030 and N1031, which increased RD3 repressive activity.

The preceding experiments measured reduced reporter protein expression by Pumilio RD3, which we previously showed corresponds to reduced reporter mRNA level (8, 9). To assess the role of the crucial RD3 phenylalanines on the ability to reduce mRNA level, we performed RT–quantitative PCR (qPCR). The results show that F1033A and F1040A mutations abolish the ability of RD3 to reduce the Nluc 2×MS2 reporter mRNA (Fig. S1).

### CR1 and CR4 of Pumilio RD3 are sufficient to cause repression

**Figure 5:**
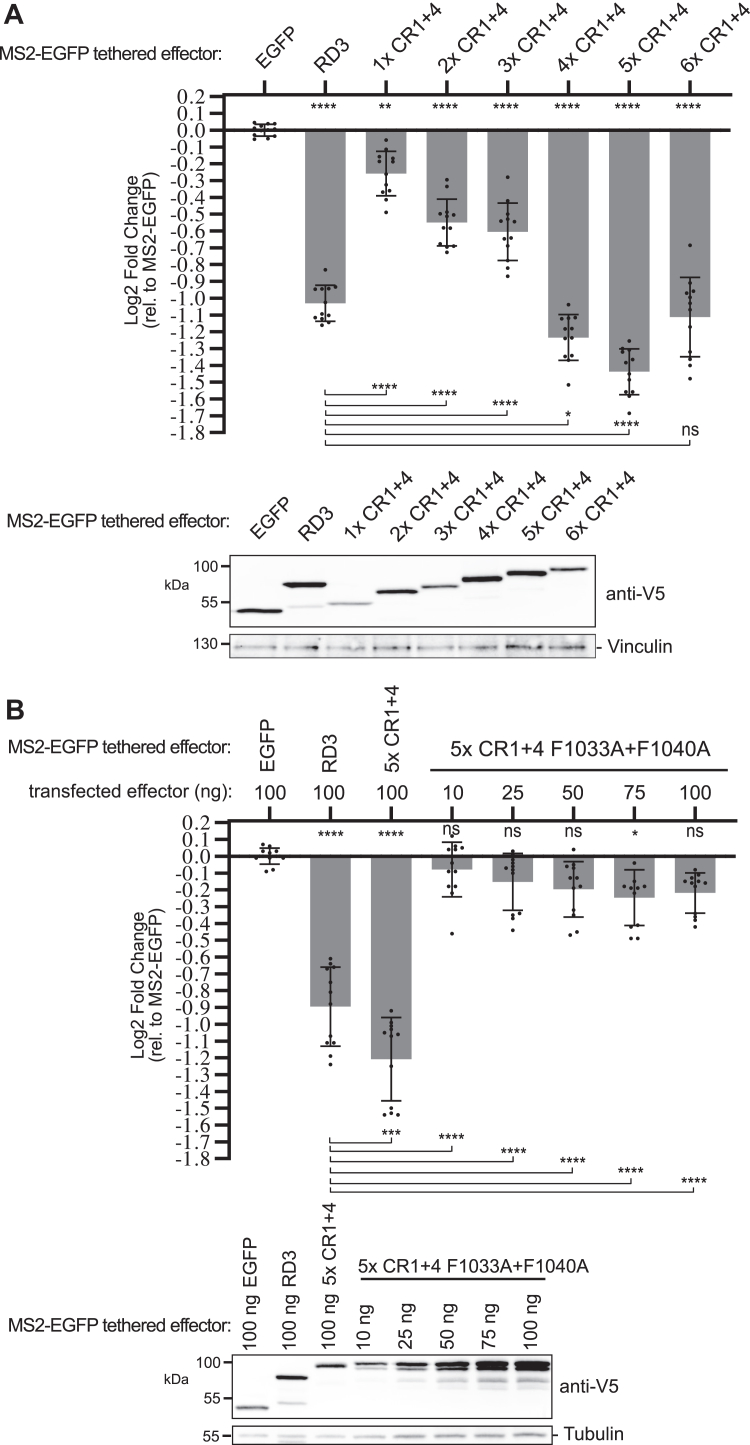
**Conserved regions CR1 and CR4 of Pumilio RD3 are sufficient to cause repression.***A*, mean and replicate log2 fold change values of CR1 + 4 multimers as measured in tethered function assays. N = 12, ±StDev. Significance is indicated *above* the axis relative to MS2-EGFP, whereas *below* the axis relative to wildtype MS2-RD3. For significance calling, *p* < 0.05 = ∗, *p* < 0.01 = ∗∗, *p* < 0.001 = ∗∗∗, and *p* < 0.0001 = ∗∗∗∗ based on ordinary one-way ANOVA and Tukey's test for multiple comparisons. All data, including the plotted replicate and mean values and statistics, are reported in Table S1. Western blot confirmed the expression of each V5-tagged test protein, as shown at the *bottom*. Vinculin served as a loading control. *B*, mean and replicate log2 fold change values of the 5× CR1 + 4 multimer in comparison to the mutant version with F1033A and F1040A substitutions, as measured in tethered function assays. The amount of transfected mutant 5× CR1 + 4 plasmid was varied, as indicated. N = 12, ±StDev. Western blot analysis confirmed the expression of each test protein, as shown at the *bottom*. Tubulin served as a loading control.

Having observed that CR1 and CR4 are crucial for RD3 activity, we tested whether they are sufficient. Our analysis of RD3 amino acids 847 to 926 (containing CR1) and amino acids 1010 to 1090 (containing CR4) indicate that these elements do not have repressive activity on their own (Fig. 2*A*). Therefore, we tested the repressive activity of CR1 combined with CR4. The activity of one to six tandem copies of CR1 + 4, fused to MS2-EGFP, was tested using the tethered function assay. In these constructs, the CRs were linked by a flexible 28 amino acid serine–glycine linker sequence (Table S2) (23). One to three copies of CR1 + 4 exhibited repression activity, albeit at levels below wildtype RD3 (Fig. 5*A*). In contrast, four to six copies of CR1 + 4 repressed reporter expression to a greater degree than wildtype RD3. Importantly, mutation of F1033 and F1040 ablated the repressive activity in the context of five copies of CR1 + 4 (Fig. 5*B*). Of note, expression of the 5× CR1 + 4 F1033A + F1040A mutant protein exceeded that of wildtype CR1 + 4 when equivalent amounts of plasmids were transfected into the cells (100 ng each). We therefore titrated the amount of transfected plasmid encoding 5× CR1 + 4 F1033A + F1040A spanning a range of 10 to 100 ng and then compared repression activity to the standard 100 ng amount of wildtype 5× CR1 + 4 plasmid (Fig. 5*B*). The mutant 5× CR1 + 4 F1033A + F1040A was inactive throughout the range, even when expressed at an equivalent level as the wildtype 5× CR1 + 4 protein (Fig. 5*B*, compare 10–25 ng of 5× CR1 + 4 F1033A + F1040A to 100 ng of wildtype). Taken together, the results of this analysis define two key features of Pumilio RD3 that contribute to its repression activity and emphasize the importance of the conserved phenylalanines.

### Pumilio RD3 interacts with the Not1, Not2, and Not3 subunits of the CCR4–NOT complex

**Figure 6:**
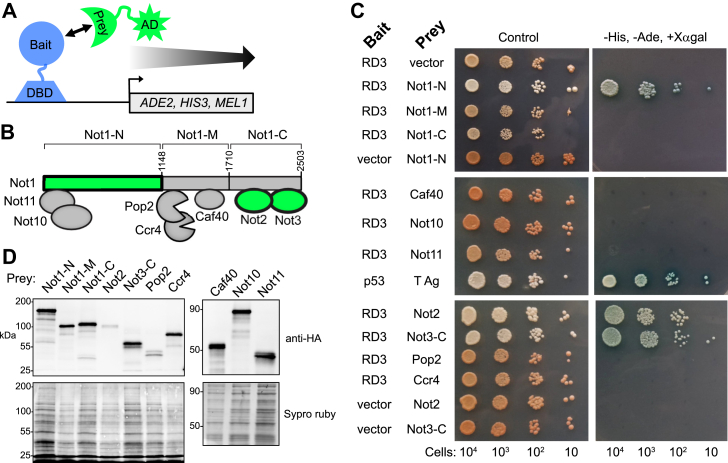
**Pumilio RD3 interacts with the Not1, Not2, and Not3 subunits of the CCR4–NOT complex.***A*, the yeast two-hybrid assay measured activation of *ADE2*, *HIS3*, and *MEL1* reporter genes in response to interaction between the Gal4 DNA-binding domain (DBD) fused to Pum RD3 protein (bait) and the prey protein fused to transcriptional activation domain (AD). *B*, subunit architecture of the *Drosophila* CCR4–NOT deadenylase complex. The portions that interact with Pumilio RD3 are highlighted in *green*. *C*, growth assays detect interactions between the indicated bait and prey proteins in the two-hybrid assay. Cells were spotted (numbers indicated at the *bottom* of *each column*) and were grown on either control medium, which selects for the bait and protein proteins, or the selective medium lacking histidine (-His), adenine (-Ade), and containing the x-ɑ-gal substrate to detect MEL1 expression *via blue colony color*. Because of the large size of Not1, prey proteins were constructed with N-terminal, C-terminal, and middle fragments, as indicated in *B*. The Not3-C prey protein contains the C-terminal region spanning amino acids 522–844. Interaction of the human p53 protein with SV40 T antigen served as a positive control. Negative controls included RD3 bait with empty prey vector. Additional negative controls included empty bait vector with the three prey proteins Not1-N, Not2, and Not3-C. *D*, Western blot analysis to detect expression of the prey proteins. Membranes were stained with Sypro Ruby to assess sample loading on the blots. Expression of bait proteins was verified by Western blot, as shown for Fig. S2.

We previously reported that RD3 interacts with, and requires the activity of, the CCR4–NOT complex. In this study, we sought to identify the specific contact(s) between RD3 and CCR4–NOT subunits. To do so, we used the yeast two-hybrid assay (Fig. 6*A*) (24). RD3 was fused to the Gal4 DNA-binding domain to create a “bait” protein. For “prey” fusion proteins, individual subunits of the *Drosophila* CCR4–NOT complex (Fig. 6*B*) were linked to the Gal4 activation domain. Bait–prey interactions were scored using three reporter genes (Fig. 6*A*), as a means to reduce false positives. Transcriptional activation of the *ADE2 and HIS3* reporters was scored by growth on media lacking adenine and histidine. In addition, on control plates, adenine auxotrophs are *pink*, whereas *ADE2* expression produces white colonies. Finally, activation of the MEL1 reporter, encoding alpha-galactosidase, was scored by *blue*-colored colonies on plates containing X-alpha-gal. As a control, we show that the RD3 bait with the empty Gal4 AD vector does not autoactivate the reporters. The positive control p53 interacts with SV40 T-antigen, as expected, resulting in growth of *blue* colonies on medium with X-alpha-gal and without histidine and adenine (Fig. 6*C*).

The large 280 kDa Not1 protein was split into three segments (Fig. 6*B*. N, M, and C) based on previous structure–function analyses, of which only Not1-N interacted with RD3 (Fig. 6*C*) (25). Two other CCR4–NOT subunits, Not2 and Not3 (C-terminal region), also interacted with RD3 (Fig. 6*C*). The other CCR4–NOT proteins did not interact with RD3 (Fig. 6*C*). In additional control tests, these bait proteins did not activate the reporters when combined with a DNA-binding domain vector that lacked RD3. Expression of each prey and bait protein was confirmed by Western blot analysis (Figs. 6*D* and S1). These observations indicate that Pumilio RD3 can interact with three subunits of CCR4–NOT: Not1, Not2, and Not3.

### Identification of a Pumilio-binding region in Not1

**Figure 7:**
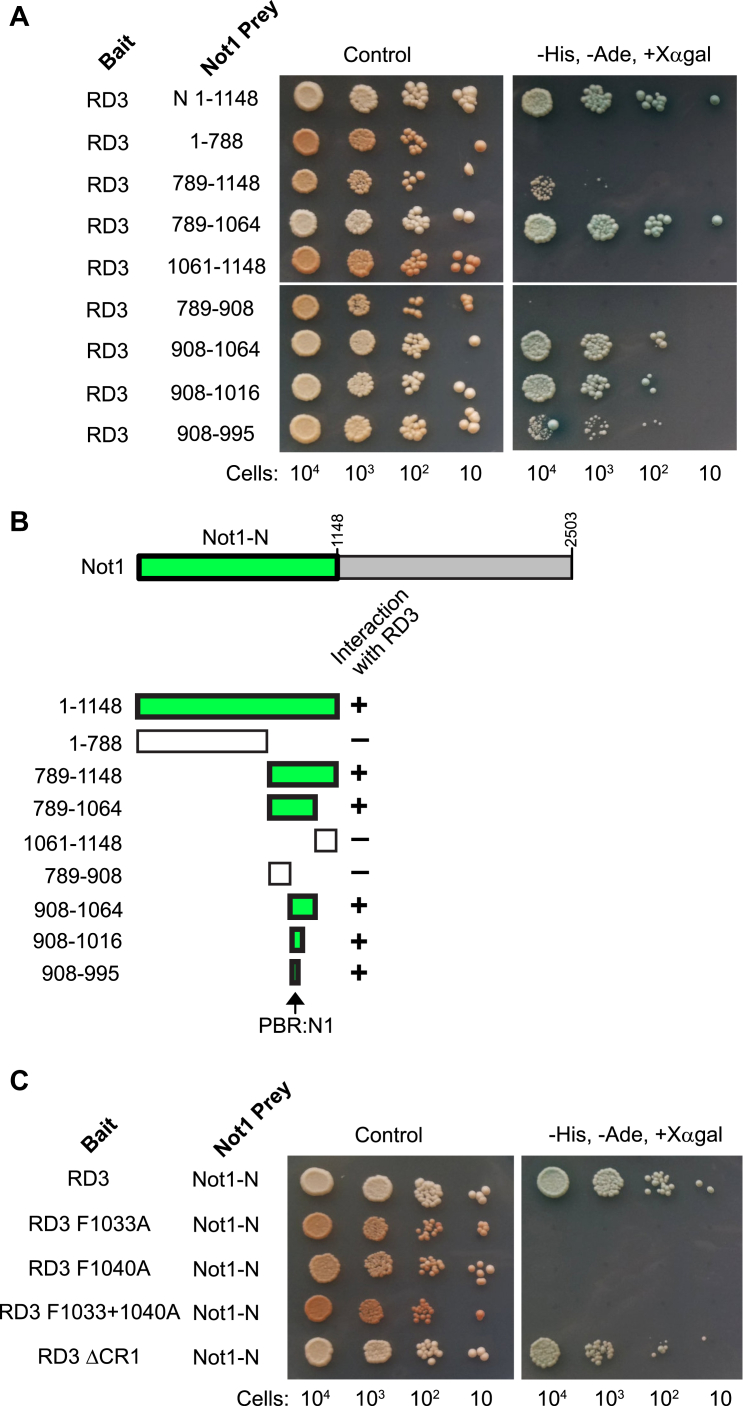
**Identification of the Pumilio RD3-binding region in the Not1 subunit of the CCR4–NOT complex.***A*, yeast two-hybrid assays with the indicated bait and prey proteins mapped the regions of Not1 that are bound by RD3, as highlighted in *green* in the diagram in *B*. The minimal Pumilio-binding region in Not1, designated PBR:N1, is indicated. *C*, interaction of wildtype or mutant versions of RD3 with Not1-N was analyzed using the yeast two-hybrid assay. Expression of bait and prey proteins was verified by Western blot, as shown for Fig. S2.

To map the region of Not1 that interacts with RD3, we performed yeast two-hybrid assays with a series of truncated Not1-N prey proteins. The results show that RD3 interacts with an 88 amino acid region of Not1-N (amino acids 908–995) within an alpha-helical MIF4G domain (Fig. 7, *A* and *B*), which we hereon refer to as the Pumilio-binding region in Not1, PBR:N1. Expression of these bait and prey proteins was confirmed by Western blot (Fig. S2). Intriguingly, this region overlaps with the portion of Not1 that is bound by the RNA-binding repressor protein Tristetraprolin (see Discussion section) (26).

Given their importance for RD3 repression activity, we examined the role of F1033 and F1040 in binding to Not1-N. We compared the interaction of wildtype and mutant versions of RD3 with Not1-N and observed that the individual and combined F1033A and F1040A mutations disrupted the RD3–Not1-N interaction, whereas deletion of CR1 did not (Fig. 7*C*). These results indicate that F1033 and F1040 of CR4 are important for the interaction of RD3 with Not1-N.

### RD3 interacts with the NOT-box regions of Not2 and Not3

**Figure 8:**
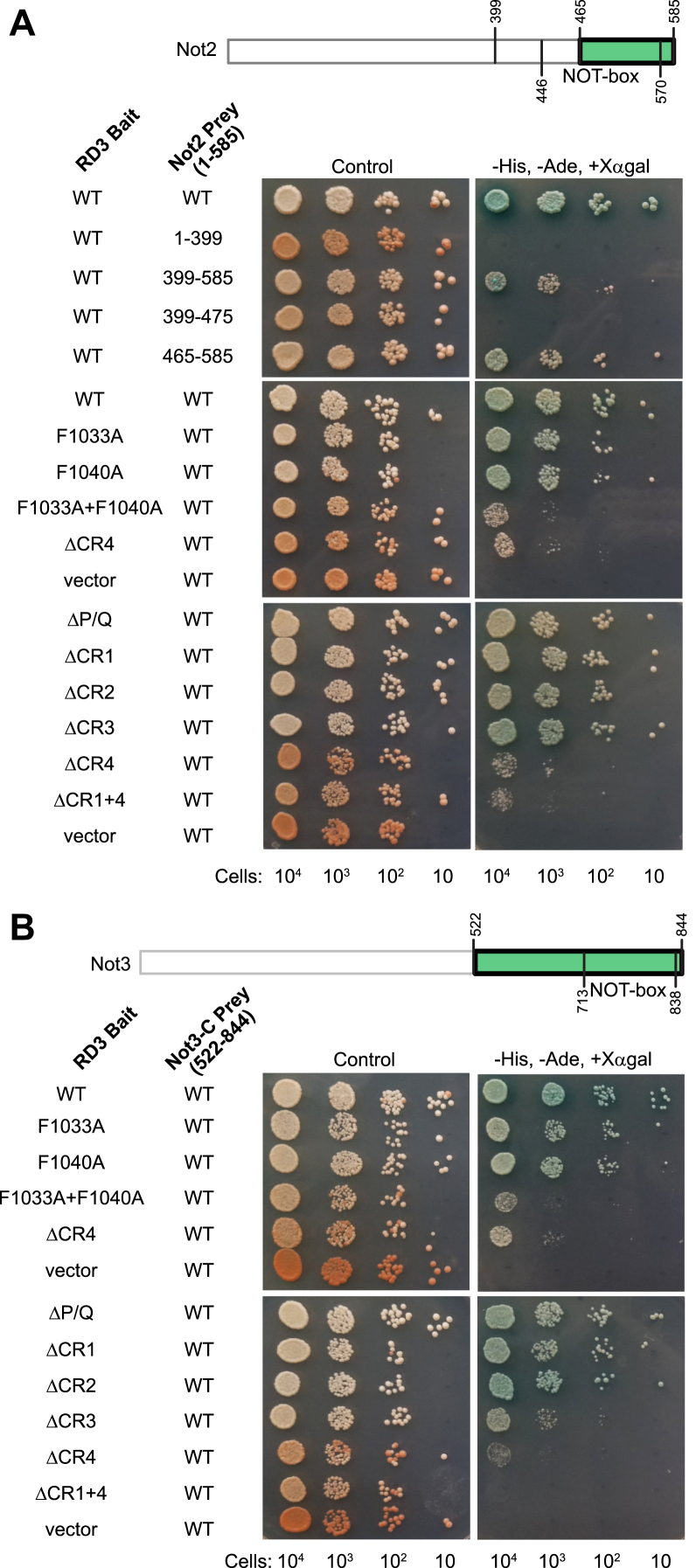
**RD3 interacts with the NOT-box regions of Not2 and Not3.** Yeast two-hybrid assays with the indicated wildtype or mutant RD3 baits and Not2 or Not3 prey proteins are shown in *A* and *B*, respectively. Regions of Not2 and Not3 proteins that interact with RD3 are shown in *green*. Expression of bait and prey proteins was verified by Western blot for Fig. S2.

We also examined the interaction of RD3 with Not2 and Not3. First, the minimal region of Not2 (amino acids 465–585) that interacts with RD3 was identified (Fig. 8*A*). This region includes the NOT-box domain (Pfam: PF04153) (27, 28), which shares homology with Not3. Individually, mutation of F1033 or F1040 had no effect on the RD3–Not2 interaction; however, when combined, growth on the selective media was diminished (Fig. 8*A*), indicative of a weakened interaction. The effects of deletion of P/Q and CR1–4 were also tested. Deletion of CR4 alone or in combination with CR1 diminished the RD3–Not2 interaction, whereas the other deletions did not (Fig. 8*A*). In a similar manner, combined F1033A and F1040A mutations diminished the interaction between RD3 and the C-terminal region of Not3 (amino acids 522–844), as did deletion of CR4 (Fig. 8*B*). In contrast to Not2, combined deletion of CR1 and CR4 prevented the RD3–Not3 interaction (Fig. 8*B*). Western blots confirmed expression of the RD3 bait and Not2 and Not3 prey proteins (Fig. S2). Together, these results indicate that the NOT-box regions of Not2 and Not3 interact with RD3. CR4 and its phenylalanine residues promote interaction of RD3 with Not2 and Not3. While CR1 is not necessary, it appears to facilitate CR4-dependent binding to Not3.

**Figure 9:**
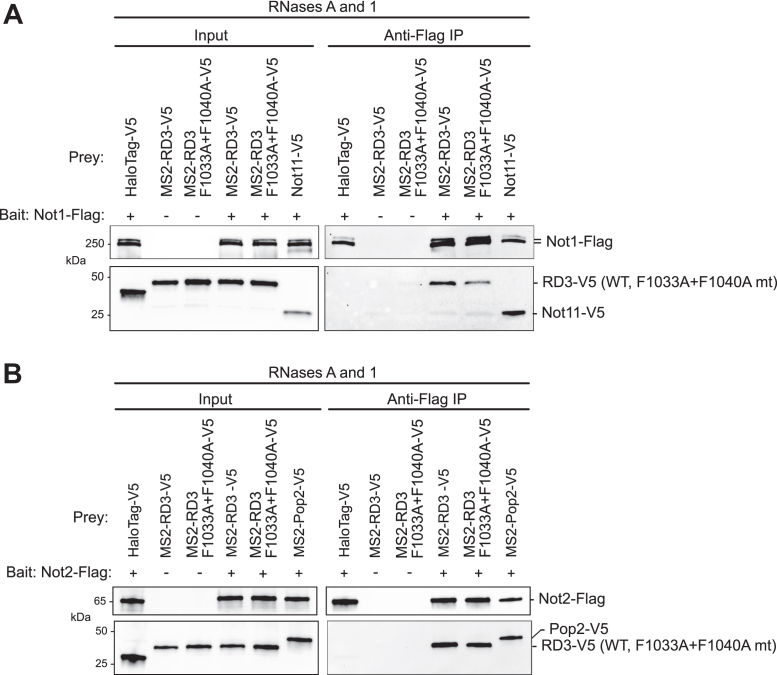
**Pumilio RD3 associates with the CCR4–NOT complex.***A*, V5-tagged wildtype or mutant (F1033A + F1040A) RD3 coimmunoprecipitate with FLAG-tagged Not1 from RNase-treated cell extracts. The 3×FLAG tag was engineered into the C terminus of the endogenous Not1 gene in the DL1 cell line. Negative control coimmunoprecipitation assays were performed from wildtype DL1 cells that do not have the FLAG tag on Not1 and were transfected with the RD3 prey constructs. Halotag-V5 served as a negative control, whereas the Not11 subunit of the CCR4–NOT complex served as a positive control. The RD3 proteins were the same MS2-fusion constructs used in the tethered function assays. *B*, the same strategy was used to detect interaction of wildtype or mutant versions of RD3 with the Not2 protein, except in this experiment, the FLAG-tagged Not2 was expressed from a cotransfected plasmid in Dmel-2 cells. V5-tagged Pop2, a component of the CCR4–NOT complex, served as a positive control. Cells cotransfected with empty bait expression vector and RD3 prey constructs served as the negative control.

Our data indicate that Not1, Not2, and Not3 converge on RD3. We next sought to analyze the interaction of RD3 with the endogenous CCR4–NOT complex by performing coimmunoprecipitation assays using cultured *Drosophila* cells. First, a 3×FLAG epitope tag was engineered onto the Not1 gene using CRISPR–Cas9. This cell line was then transfected with plasmid expressing V5 epitope–tagged versions of wildtype RD3 or the RD3 F1033A + F1040A mutant. Both wildtype and mutant RD3 coimmunoprecipitated with Not1 (Fig. 9*A*). Likewise, both wildtype and mutant RD3 associated with coexpressed FLAG-tagged Not2 in a separate experiment (Fig. 9*B*). The samples were treated with RNases, which did not prevent the interaction with Not1 or Not2. The V5-tagged Not11 (Fig. 9*A*) or Pop2 (Fig. 9*B*) components of the CCR4–NOT complex served as positive controls, whereas V5-tagged Halotag served as a negative control. Specificity of the RD3–Not1 association was also assessed by performing negative control immunoprecipitation assays from wildtype cells that lacked tagged versions of Not1 or Not2 (Fig. 9, *A* and *B*). The results indicate that mutation of the F1033 and F1040 does not prevent RD3 from interacting with the endogenous CCR4–NOT complex. Thus, while the phenylalanine residues are needed for interaction with Not1-N, the remaining contacts of RD3 with Not2 and Not3 are sufficient to maintain the association with CCR4–NOT. Together with the functional data showing that F1033 and F1040 are necessary for repression activity, these results indicate that contact with Not1-N is of particular importance to RD3-mediated repression.

### Role of RD3 in Pumilio-mediated repression of target mRNAs

**Figure 10:**
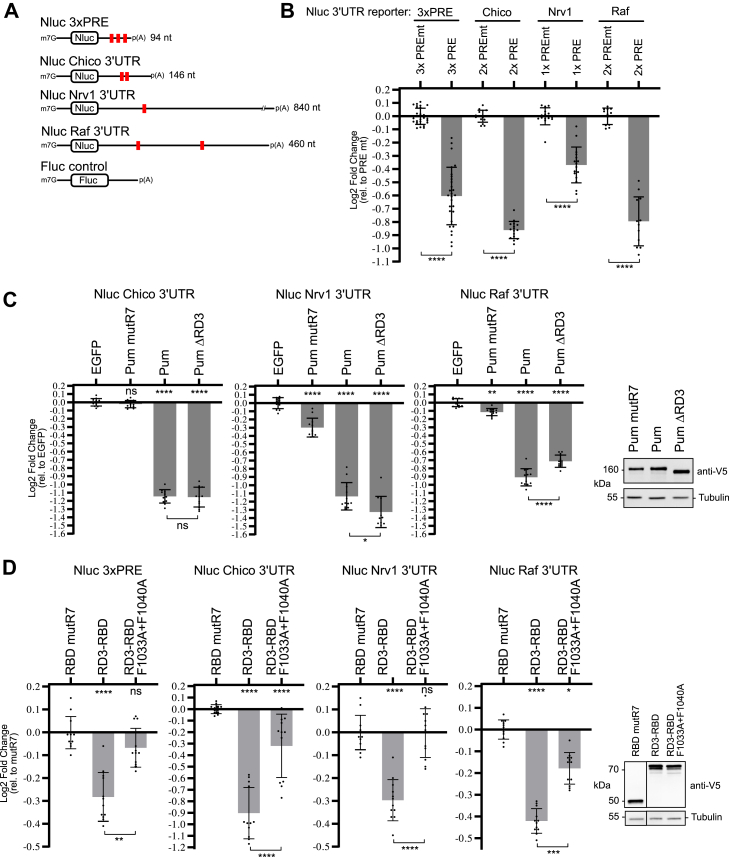
**Role of RD3 in Pumilio-mediated repression of target mRNAs.***A*, nano-luciferase–based reporter genes include Nluc 3×PRE with three copies of the PRE in a minimal 3′UTR, or the PRE-containing 3′UTRs from the Chico, Nrv1, and Raf mRNAs. PREs are indicated by *red boxes*. For each reporter, a corresponding PRE mutant (PREmt) version that is not bound by Pumilio was created by mutating the 5′-UGU of the PRE to 5′-ACA. Fluc reporter gene served as an internal control. *B*, mean and replicate log2 fold change values were measured for each wildtype Nluc reporter gene relative to its corresponding PRE mutant control, in dual luciferase assays. In this assay, endogenous Pumilio represses the PRE-containing reporter in DL1 cells. N ≥ 12, ±StDev. Significance is indicated *above* the axis relative to the PRE mt version of each reporter. For significance calling, *p* < 0.05 = ∗, *p* < 0.01 = ∗∗, *p* < 0.001 = ∗∗∗, and *p* < 0.0001 = ∗∗∗∗ based on two-tailed unpaired Student’s *t* test. All data, including the plotted replicate and mean values and statistics, are reported in Table S1. *C*, using DL1 cells, repression of the indicated reporters by transfected wildtype Pumilio (Pum) was compared with a construct wherein RD3 is deleted (Pum ΔRD3). Mean and replicate log2 fold change was calculated relative to the negative control MS2-EGFP. The Pum mutR7, an RNA-binding defective mutant version of Pum, served as an additional negative control. N = 12, ±StDev. For significance calling, *p* < 0.05 = ∗, *p* < 0.01 = ∗∗, *p* < 0.001 = ∗∗∗, and *p* < 0.0001 = ∗∗∗∗ based on ordinary one-way ANOVA and Tukey's test for multiple comparisons. Expression of the V5-tagged test proteins was confirmed by Western blot analysis, as shown on the *right*. Tubulin served as a loading control. *D*, repression by a transfected RD3-RBD test protein was tested in comparison to the F1033A + F1040A mutant version in Pumilio knockout DL1 cells. Mean and replicate log2 fold change was calculated relative to the RNA-binding defective mutR7 RBD. N = 12, ±StDev. For significance calling, *p* < 0.05 = ∗, *p* < 0.01 = ∗∗, *p* < 0.001 = ∗∗∗, and *p* < 0.0001 = ∗∗∗∗ based on ordinary one-way ANOVA and Tukey's test for multiple comparisons. Expression of the V5-tagged test proteins was confirmed by Western blot analysis, shown on the *right*. PRE, Pumilio response element.

We sought to test the role of RD3 CR4 and its phenylalanine residues in Pumilio-mediated repression of target mRNAs. To do so, we used Nluc-based reporter mRNAs under the control of the 3′UTRs of naturally occurring target mRNAs, *Chico*, *Nrv1*, and *Raf* (Fig. 10*A*). We identified these target mRNAs based on their content of one or more consensus PRE (3) and experimental evidence that supports Pumilio binding, as detected by RNA coimmunoprecipitation analysis (29). As a positive control, we included a reporter with a minimal 3′UTR containing three PREs that is efficiently repressed by Pumilio (Fig. 10, *A* and *B*; Nluc 3×PRE) (8). First, we demonstrated the repressive activity of the PREs in wildtype *Drosophila* cells. Each PRE-containing reporter was significantly repressed relative to the same reporter bearing PRE mutations (5′-UGUANAUA to 5′-ACAANAUA) that prevent binding by Pumilio (Fig. 10*B*) (4, 5, 9).

Next, we tested the ability of overexpressed wildtype Pumilio, or a mutant version wherein RD3 has been deleted (Pum ΔRD3), to repress each reporter relative to negative-control EGFP in DL1 cells. Wildtype Pumilio robustly repressed each reporter, whereas an RNA-binding defective mutant Pumilio (Pum mutR7) had minimal effect (Fig. 10*C*), consistent with previous observations (5, 8, 9, 14). Deletion of RD3 did not prevent repression of *Chico* and *Nrv1* reporters by Pumilio but did diminish repression of the *Raf* reporter (Fig. 10*C*). These observations indicate that RD3 is not essential for repression by Pumilio, which agrees with published evidence that the three N-terminal RDs exhibit functional redundancy and that removal of a single RD does not eliminate activity (8, 9).

To determine the role of the Pumilio RD3 CR4 phenylalanines in repression of PRE-containing mRNAs, we created a test protein that contains RD3 and the RBD of Pumilio (RD3-RBD, amino acids 847–1533) by deleting the region spanning RD1-PCMb of Pumilio (Fig. 1). Repression activity of wildtype RD3-RBD was compared with a mutant version wherein both F1033 and F1040 were substituted by alanines. To avoid potential competition of the test proteins with endogenous Pumilio, we created a *Drosophila* cell line wherein the *Pumilio* gene was knocked out using CRISPR–Cas9 (Fig. S3). The RD3-RBD protein repressed each reporter effectively; however, mutation of F1033 and F1040 significantly reduced (*i.e.*, 3×PRE, *Chico*, and *Raf* reporters) or eliminated repression (*i.e.*, *Nrv1* reporter) (Fig. 10*D*). These observations demonstrate the key roles of Pumilio RD3 CR4 phenylalanines in repression.

## Discussion

The mechanisms of mRNA regulation are an area of intensive investigation that is revealing how *cis*-acting RNA elements and *trans*-acting regulators control translation, stability, and localization of transcripts (1, 30, 31, 32). The results of this study further our understanding of how an archetypal sequence-specific regulator, Pumilio, controls the fate of its target mRNAs (3, 16).

We identified CRs of Pumilio RD3 that are important for its repressive activity. In particular, CR1 and CR4 are necessary and sufficient to elicit repression (Figure 2, Figure 3, Figure 4, Figure 5), with two phenylalanine residues (F1033 and F1040) in CR4 being of particular importance. The CR3 and P/Q regions also contribute to RD3 activity (Fig. 2). Building upon our previous work showing that RD3 associates with the CCR4–NOT complex (8), we now demonstrate that RD3 interacts with the Not1, Not2, and Not3 subunits (Figure 6, Figure 7, Figure 8). These contacts, along with the functional dependence of RD3 repression activity on Not1, Not2, Not3, and the Pop2 deadenylase subunits (8), reveal the important role of RD3-mediated recruitment of CCR4–NOT to repress target mRNAs.

We mapped the contacts between Pumilio RD3 and CCR4–NOT. RD3 binds to a specific region in the N-terminal MIF4G portion of Not1 (Fig. 7, PBR:N1). RD3 also contacts the C-terminal regions of Not2 and Not3, which include conserved NOT-Box homology regions that mediate Not2–Not3 heterodimer formation, and protein motifs that interact with Not1-C (Fig. 6*B*) (27, 28, 33). These contacts may be evolutionarily conserved because RD3 regions from the human Pumilio orthologs, PUM1 and PUM2, were recently shown to bind to a recombinant purified CCR4–NOT complex (17). It is important to acknowledge the possibility that Pumilio RD3 might make additional contacts with CCR4–NOT that escaped detection by the yeast two-hybrid assays.

In theory, the Pumilio RD3–Not1, RD3–Not2, and RD3–Not3 interactions might occur independently; however, in the context of the native CCR4–NOT complex, Pumilio RD3 is likely to simultaneously contact the three CCR4–NOT subunits. This convergence of contacts implies that the Not1-N region may be in close proximity to the Not2–Not3 dimer bound to the C-terminal region of Not1. The 3-dimensional structures of the *Drosophila* CCR4–NOT complex and Pumilio remain to be determined, and future structural analysis will be necessary to fully understand their contacts and conformation.

Our observations support a model wherein Pumilio RD3 CR4 functions as a short linear interaction motif that binds to CCR4–NOT. CR1 facilitates CR4 function, suggesting that it may make additional contacts with CCR4–NOT or stabilize CR4 conformation. Deletion of CR1 and CR4 reduced RD3 repression activity and also prevented (*i.e.*, Not3) or reduced (*i.e.*, Not2) the protein–protein interactions detected by yeast two-hybrid assays (Figure 3, Figure 7, and 8. Moreover, mutation of the two phenylalanines in CR4 eliminated RD3 repression activity and either eliminated (*i.e.*, Not1) or reduced (*i.e.*, Not2, Not3) the protein–protein contacts (Figure 4, Figure 7, and 8). The fact that the RD3 F1033A + F1040A mutant can still associate with CCR4–NOT in coimmunoprecipitation assays from *Drosophila* cells (Fig. 9) indicates that remaining interactions with Not2 and Not3 are sufficient to maintain the association. The loss of repression and Not1-binding activities of RD3 F1033A + 1040A emphasizes the crucial function of the PBR:N1 contact and leads us to speculate that it may induce a conformational, allosteric, or compositional change in CCR4–NOT that is necessary for repression.

The interaction of Pumilio RD3 with Not1 bears a striking similarity to that of another sequence-specific RNA-binding protein, Tristetraprolin (TTP/ZFP36) (34), also known as Tis11 in *Drosophila* (35). Like Pumilio, Tis11 interacts with CCR4–NOT to degrade target mRNAs (36, 37). Analogous to Pumilio RD3, the C terminus of TTP binds to the Not1 N-terminal MIF4G region (26), mediated by a 15 amino acid TTP peptide with a conserved phenylalanine residue that interacts with a hydrophobic groove of the MIF4G domain of Not1 (26, 34). These observations suggest that Pumilio and Tis11 may compete, or perhaps collaborate, to recruit CCR4–NOT to target mRNAs.

In the context of full-length Pumilio, with its four repressive domains (9, 14), the network of protein–protein contacts with CCR4–NOT is likely to be more complex. Indeed, previous studies showed that RD1, RD2, RD3, and the RBD of Pumilio can interact with CCR4–NOT (8, 14, 15). We anticipate that each of the RDs has its own unique motifs that bind to specific CCR4–NOT subunits. The resulting multivalent interactions may contribute to the affinity and specificity of the interaction to promote efficient recruitment of the deadenylase complex to target mRNAs by Pumilio. Precedence for such a model was established for the microRNA RNA-induced silencing complex, wherein GW182 makes multiple contacts with CCR4–NOT (38).

Our results reveal three new Pumilio-regulated mRNAs: *Chico*, *Raf*, and *Nrv1*. The functions of these target genes allude to the potential importance of their regulation by Pumilio. *Raf* encodes a serine–threonine kinase proto-oncogene that regulates cell proliferation. Like Pumilio, *Raf* has functions in germline, development, and memory formation (39). *Chico* encodes a substrate of the insulin receptor signaling pathway that regulates cell growth and size, development, life span, fertility, and learning (40, 41, 42, 43). *Nrv1* (*Nervana 1*) encodes a subunit of the Na+ K+ ATPase ion pump that maintains cellular membrane potential (44). Future research will be necessary to establish the biological significance of the Pumilio PRE-mediated regulation of these important genes.

## Experimental procedures

### Cell culture

*Drosophila* Dmel-2 cells (Invitrogen) were cultured at 25 °C in SF900III (Gibco) medium with no additional supplements. *Drosophila* DL1 cells (Drosophila Genomics Resource Center) were grown at 25 °C in Schneider’s *Drosophila* medium (SDM; Gibco) supplemented with glutamine (1× GlutaMAX; Gibco), 1× antibiotic–antimycotic containing 100 units/ml of penicillin, 100 μg/ml of streptomycin, and 0.25 μg/ml of amphotericin B (Thermo Fisher Scientific), and 10% heat-inactivated fetal bovine serum (GenClone).

### Luciferase reporter gene assays

Luciferase-based assays were performed using previously established conditions and reporter genes (8, 9, 14). For tethered function assays, Dmel-2 cells were transfected with pAC5.4 Nluc2 2×MS2BS reporter and pAC5.1 Fluc2 minimal 3′UTR plasmid (8). The test proteins were expressed from cotransfected plasmids (as indicated in the figures) as a fusion to the RNA-binding MS2 phage coat protein, as previously established (8, 9, 14). These test proteins were based on the plasmid vector pIZ MS2 and include a C-terminal V5 epitope tag for Western blot detection. The negative control, pIZ MS2-EGFP, was previously described (8, 9). Table S2 provides a list of plasmids used in this study.

Pumilio PRE-mediated repression assays were performed in DL1 cells that were transfected with the Nluc-based reporter plasmid, as indicated in Figure 10, along with the pAC5.1 Fluc2 minimal 3′UTR plasmid. The pAC5.4 Nluc 3×PRE plasmid contained three copies of the wildtype PRE within a minimal 3′UTR in the pAC5 vector (Invitrogen). To create the *Chico*, *Raf*, and *Nrv1* reporters, the 3′UTRs of each gene, spanning the first nucleotide after the stop codon to the last nucleotide preceding the cleavage and polyadenylation element, were inserted into the Xho1 and Not1 sites in the pAC5.4 Nluc plasmid. Pumilio test proteins, or either the EGFP or Pumilio mutR7 (S1342A, N1343A, and E1346A) controls, were expressed from plasmid pIZ as previously described (8, 9, 14).

For reporter assays, 1.5 × 10^5^ cells were seeded in 100 μl per well of a 96-well plate. Four wells per condition served as independently transfected biological replicates for luciferase measurements. An additional three wells per condition were transfected and used for subsequent Western blot analysis, as described later. Standard transfection conditions in DL1 cells included 1 ng of Fluc plasmid, 1 ng of Nluc reporter, and 98 ng of test protein expression plasmid per well. Unless indicated otherwise, Dmel-2 transfections included 5 ng Fluc, 5 ng Nluc, and 90 ng of test protein expression plasmid. For titration experiments, the empty expression vector, pIZ, was added to keep the total amount of transfected plasmid equivalent across conditions. Fugene HD transfection reagent (Promega) was used at a 4:1 v/w ratio of Fugene:DNA (0.4 μl:0.1 μg well) in serum-free media. Luciferase activities were measured 48 h after transfection using Nano-Glo dual reporter assay according to the manufacturer’s protocol (Promega) in a GloMax Discover luminometer.

The luciferase assay data were analyzed as previously described (8, 9, 21). The Nluc activity (in relative light units) for each sample was divided by its corresponding Fluc activity to calculate a relative response ratio. Repression activity of each test protein was determined as the fold change of relative response ratio relative to that of the negative control condition: MS2-EGFP for tethered function assays (Figure 2, Figure 3, Figure 4, Figure 5); PRE mutants for PRE reporter assays (Fig. 10*B*), or EGFP or Pumilio mutR7 for PRE reporter assays (Fig. 10, *C* and *D*). All results were verified in at least three independent experiments, each with four biological replicates. All data and statistics are reported in Table S1. Statistical analysis for luciferase assays was performed using ordinary one-way ANOVA using GraphPad Prism, version 9 (GraphPad Software, Inc), as noted the in the figure legends and Table S1. Multiple comparisons were made using the Tukey's test. In Figures 10*B* and S1, the statistical analyses used a two-tailed unpaired Student’s *t* test. Significance calling was based on the convention: *p* < 0.05 = ∗, *p* < 0.01 = ∗, *p* < 0.001 = ∗∗∗, and *p* <0.0001 = ∗∗∗∗.

### Western blot analysis

Cells (∼5 × 10^5^ per condition) were harvested by centrifugation at 900*g* for 4 min and lysed in radioimmunoprecipitation buffer (25 mM Tris–HCl [pH 7.6], 1 mM EDTA, 1% NP-40, 1% sodium deoxycholate, and 0.1% SDS) containing 2× cOmplete Protease Inhibitor Cocktail (Roche). Lysates were then cleared of cellular debris by centrifugation at 21,000*g* for 10 min. Protein concentration of the supernatant was measured using the detergent-compatible protein assay kit according to the manufacturer’s directions (Bio-Rad). For each sample, 10 μg of total protein extract was incubated with an equal volume of 2× SDS loading buffer at 85 °C for 10 min. Samples were then separated by SDS-PAGE and blotted to Immobilon P membranes (Millipore). After blocking the membrane for 1 h, the primary antibody (as indicated in the figures) was applied for 1 h at room temperature, or overnight at 4 °C, with gentle rocking. Antibodies, their dilution factor, and buffer condition are listed later. Membranes were washed three times for 10 min, then horseradish peroxidase (HRP)–linked secondary antibody was applied for 1 h at room temperature. After three washes of 10 min each, chemiluminescent substrate (Thermo or Millipore) was added to the membrane, which was then imaged with a ChemiDoc Touch instrument (Bio-Rad).

### Antibodies

The following antibodies were used for Western blot analysis at the indicated dilutions in either blotto (1× PBS containing 10 mM Na_2_HPO_4_ and 1.8 mM KH_2_PO_4_ at pH 7.4, 137 mM NaCl, 2.7 mM KCl, 0.1% Tween-20, and 5% w/v nonfat powdered milk) or TBST (1× Tris–HCl buffered saline containing 50 mM Tris–HCl, pH 7.5, 150 mM NaCl, 0.1% Tween-20, and 5% w/v bovine serum albumin), as specified by the antibody’s manufacturer.

Mouse anti-V5 primary antibody (Invitrogen; catalog no.: R960-25) at 1:5000 dilution in blotto.

Rabbit anti-V5 (Cell Signaling Technologies; catalog no.: 13202S) at 1:5000 dilution in TBST.

Mouse anti-Tubulin (Cell Signaling Technologies; catalog no.: 3873) at 1:1000 dilution in blotto.

Rabbit anti-Vinculin (Thermo Fisher Scientific; catalog no.: 700062) at 1:1000 dilution in blotto.

Rabbit anti-Myc (Cell Signaling Technologies; catalog no.: 2278S) at 1:5000 dilution in TBST.

Rabbit antihemagglutinin (HA) (Cell Signaling Technologies; catalog no.: 3724s) at 1:5000 dilution in TBST.

Rabbit anti-FLAG (Sigma; catalog no.: sab4301135) at 1:5000 dilution in blotto.

Mouse anti-FLAG (Sigma; catalog no.: F3165-1MG) at 1:5000 dilution in blotto.

Goat anti-rabbit-HRP secondary antibody (Cell Signaling Technologies; catalog no.: 7074P2 or Sigma, catalog no.: AP187P) at 1:5000 dilution in blotto.

Goat antimouse-HRP (Thermo Fisher Scientific; catalog no.: 31430) at 1:5000 dilution in blotto.

### Yeast two-hybrid assays

The yeast two-hybrid assays used the *Saccharomyces cerevisiae* Y2H Gold strain (Takara Bio) with genotype: *MATa*, *trp1-901*, *leu2-3*, *112*, *ura3-52*, *his3-200*, *gal4Δ*, *gal80Δ*, *LYS2 : : GAL1UAS–Gal1TATA–His3*, *GAL2UAS–Gal2TATA–Ade2*, *URA3 : : MEL1UAS–Mel1TATA*, *AUR1-C MEL1.* Bait and prey proteins were expressed using pGBKT7 and pGADT7 vectors, respectively (Takara Bio). Plasmid transformations of the yeast were made using the lithium acetate–based method according to the manufacturer’s protocol (Takara Bio) with 1 μg of pGBKT7- and pGADT7-based plasmids. To select transformants, yeasts were grown on double dropout (DDO) plates with medium lacking leucine and tryptophan for 3 days at 30 °C. For growth assays, colonies from each DDO plate were inoculated in 5 ml DDO synthetic dextrose medium and grown overnight at 30 °C. The following day, 1 ml of culture was taken and grown in 4 ml medium until the absorbance reached 1.0 at 600 nm. Each culture was serially diluted from 2 × 10^6^ cells/ml to 2 × 10^3^ cells/ml, and 5 μl of each dilution was plated as spots on DDO and quadruple dropout (-Leu, -Trp, -His, -Ade, QDO) plates supplemented with 100 μl of 4 μg/ml X-alpha-gal (5-bromo-4-chloro-3-indolyl α-d-galactopyranoside). Plates were incubated at 30 °C and then photographed. Each interaction was tested in three experimental replicates.

For Western blot detection of Myc-tagged bait and HA-tagged prey proteins, each cotransformant was grown to a cell density (absorbance at 600 nm) of 0.7, and 10 million cells were harvested by centrifugation. Cell extracts were then prepared by adding 75 μl of radioimmunoprecipitation buffer with 4× cOmplete Protease Inhibitor Cocktail (Sigma) and 1 mM PMSF and bead bashing with glass beads in a Disruptor Genie (Scientific Industries) five times at 1 min intervals at 4 °C. Cellular debris was cleared by centrifugation at 14,000*g* for 5 min in a microfuge. Next, 10 μl of 5× SDS loading dye was added to the supernatant, and samples were incubated at 85 °C for 10 min. Cell extracts, 10 μl each, were then analyzed by SDS-PAGE and Western blotting. Total protein on the Immobilon polyvinylidene difluoride membranes (Millipore) was detected by staining with Sypro Ruby (Thermo Fisher Scientific) following the manufacturer’s directions and imaged on a Chemidoc Touch (Bio-Rad). Western blot signals were detected using anti-Myc and anti-HA primary antibodies and HRP-conjugated secondary antibodies with enhanced chemiluminescence (Thermo Fisher Scientific) and a ChemiDoc Touch.

### Generation of DL1 Not1-3×FLAG cell line

A 3×FLAG tag was engineered onto the C terminus of the Not1-coding region (Fig. S3*A*) using CRISPR–Cas9 in the DL1 *Drosophila* cell line. Guide RNA sites targeting exon 10, which is present in Not1 isoforms PC, PD, PE, PH, and PG, were designed using Benchling CRISPR RNA guide software. The single-guide RNA plasmid pAc sgRNA Cas9 Not1 was created by inserting annealed primers RJH 212 Not1 Sg1 (forward: 5′-ttcgCTCCAGGCAGAAGCGTCGGA) and RJH 213 Not1 SG1 (reverse: 5′-aacTCCGACGCTTCTGCCTGGAGc) into the BspQ1 site (45). Note that the 5′ “ttc” or “aac” (in lowercase) are BsqQI cohesive overhangs, and the g:c base pair was included as part of the U6 promoter. A single-stranded homology–directed repair donor template (IDT) containing Not1 homology arms (in capital letters), a tobacco etch virus protease cleavage site (in italics), a 3×FLAG tag (in lowercase letters), and in-frame stop codon (bold, capital letters) was created with the following sequence: Not1 tobacco etch virus 3×FLAG ssODN: 5′-CGTCGATGGCGAGGGCCAGGAGGTAGCCACCATCAACctt*gaggatctgtactttcagagc*cttgactacaaagaccatgacggtgattataaagatcatgacatcgattacaaggatgacgatgacaag**TGA**ATGGATCCACGTCCGACGCTTCTGCCTGGAGTTCTGCGCGAGACCCAGACGCAGGCAGTAGCTGCCT.

DL1 cells (2 × 10^6^ per well) were plated in 2 ml of SDM in a 6-well plate. After 24 h, cells were transfected with 4 μl FuGene HD (Promega), 1 μg sgRNA-Cas9 plasmid DNA, 40 pmol ssODN template, and 100 μl serum-free SDM. After incubation for 48 h at 25 °C, the medium was replaced with fresh SDM containing 5 μg/ml puromycin (Gibco). About 72 h later, when cells approached confluence, they were expanded into a 10 cm dish and allowed to continue growing to 80% density again. Clonal lines were then isolated by limiting dilution. The Not1-3×FLAG cell line was identified by Western blot detection with anti-FLAG (Fig. 9) and confirmed by PCR amplification and sequencing of Not1 exon 10 from genomic DNA (Fig. S3*A*).

### Coimmunoprecipitation analysis

DL1 cells, Not1-3×FLAG DL1 cells, or Dmel-2 cells (3 × 10^6^ per well) were seeded in a 6-well plate in 2 ml of SDM (DL1 cells) or SF900III (Dmel-2 cells) and grown for 24 h at 25 °C. The cells were then transfected using Fugene HD reagent at a 4:1 v/w ratio of Fugene HD:DNA (12 μl:3 μg well) in serum-free media with either 3 μg total of prey protein expression plasmid (Fig. 9*A*) or 1.5 μg each of bait and prey protein expression plasmids (Fig. 9*B*). The prey proteins were expressed from the pIZ plasmid vector with C-terminal V5 epitope tag (Invitrogen) and included wildtype or mutant (F1033A and F1040A) versions of MS2-RD3-V5. Plasmids expressing Halotag-V5 or Not11-V5 served as the negative or positive control, respectively. The bait protein Not2-3×FLAG was expressed from plasmid pIZ Not2-3×FLAG. Transfected cells were incubated at 25 °C and, after 3 days of growth, were harvested. Cells were washed once with 1× PBS, then incubated on ice for 10 min in lysis buffer (50 mM Tris–HCl [pH 8.0], 500 mM NaCl, 1 mM EDTA, 0.2% Triton X-100, and 2× cOmplete Protease Inhibitor Cocktail). Cells were mechanically disrupted with a pellet pestle (Fisher Scientific) for 20 s each, and then lysates were centrifuged at 21,000*g* for 10 min in a microcentrifuge. The supernatant was then passed through a spin column with 0.45 μm cutoff (Millipore).

EZview Red anti-FLAG M2 Affinity Gel (Sigma), 10 μl bed volume per sample, was prepared by washing four times with wash buffer (50 mM Tris–HCl [pH 8.0], 500 mM NaCl, and 1 mM EDTA). The supernatants were then added to the anti-FLAG beads in a 700 μl volume containing RNases One (10 units) and A (4 μg)(Promega). Binding was performed with end-over-end rotation at 4 °C for 3 h. The beads were then collected by centrifugation at 4000*g* for 1 min, and the supernatant was removed. The beads were washed three times with 1 ml of lysis buffer without protease inhibitors for 5 min with end-over-end rotation at 4 °C, followed by three washes with wash buffer. Bound proteins were eluted from 20 μl of immunoprecipitation pellets by adding 20 μl 2× SDS loading dye and incubating at 85 °C for 10 min. The samples were then analyzed by SDS-PAGE and Western blot using rabbit anti-V5 and anti-FLAG antibodies to detect prey and bait proteins, respectively. Western blots included “input” cell lysates (10 μg total protein, as determined by detergent-compatible Lowry assay; Bio-Rad).

### Knockout of Pumilio in DL1 cells

Pumilio was knocked out in the DL1 cell line using CRISPR–Cas9 genome engineering to introduce indels. The *Drosophila* Pum gene is located on the third chromosome and, according to annotations in the National Center for Biotechnology Information and Flybase databases, encodes eight transcripts, Pum RA-RH, that encode isoforms Pum PA-PH. The longest isoform encodes the canonical Pumilio protein, Pum-PA (Fig. 1). Guide RNA sites in Pumilio were identified using Benchling CRISPR RNA guide software, and guide RNAs were designed to target exon 9, the first exon that is common to all Pumilio isoforms (Fig. S3*B*). The sequence of the guide RNA targeting site was confirmed by PCR amplification from DL1 genomic DNA and Sanger sequencing (Fig. S3, *C* and *D*). DNA oligos encoding the sgRNA sequence were cloned into the BspQI sites in the pAc-sgRNA-Cas9 vector (45) using primers RJH528 Pum exon 9 (forward: 5′-ttcGCAACAACAGATGCACATGG) and RJH529 Pum exon 9 (reverse: 5′-aacCCATGTGCATCTGTTGTTGC) to generate plasmid RJH247 pAc sgRNA Cas9 dPum sg2. The plasmid was then transfected into DL1 cells that were plated at a density of 1 × 10^6^ per milliliter in 2 ml complete media in a 6-well dish. Transfection mixes contained 8 μl FuGene HD (4:1 ratio), 2 μg sgRNA-Cas9 plasmid DNA, and 1 ml serum-free SDM following the manufacturer’s protocol (Promega). Cells were incubated at 25 °C for 48 h, and then media were removed and replaced by selective media containing 5 μg/ml puromycin. Knockout clonal lines were then isolated by limited dilution. The clonal Pumilio knockout lines were analyzed by Inference of CRISPR Edits from Sanger Trace Data (ICE, Synthego). A homozygous knockout clonal line was identified and confirmed by sequencing exon 9 from genomic DNA, which was amplified by PCR using primers RJH 191 Pum exon 9 (forward primer: 5′-AACTGTTTCGCTCGCAGAATCCG) and RJH 192 Pum exon 9 (reverse primer: 5′-TGATACGGCTGATTCTCGGCACC). We identified a clonal line with homozygous 10 bp deletion in Pum exon 9, which causes a frameshift after methionine 726 that creates a truncated 765 amino acid protein that would be nonfunctional because of the lack of RBD (Fig. S3, *C*–*E*). In addition, the mRNA produced would be subject to nonsense-mediated mRNA decay, consistent with our observation of reduced *Pumilio* mRNA in the knockout cells relative to wildtype (Fig. S3*F*).

### RT and qPCR

For analysis of *Pumilio* mRNA in Fig. S3*F*, total RNA was purified from wildtype and Pumilio knockout DL1 cells using Maxwell RSC simply RNA tissue kit with on-bead DNase I digestion to remove genomic DNA (Promega). Reverse transcription was then performed using GoScript reverse transcriptase (Promega) with 5 μg of total RNA and random hexamer primers according to the manufacturer’s protocol. The complementary DNA (cDNA) was then diluted with 100 μl water to a final concentration of 40 ng/μl. As a negative control, equivalent reactions were performed in the absence of the RT enzyme (no RT control). qPCR was then performed using Go-Taq qPCR Master Mix (Promega) containing 2 μl of cDNA sample in a 20 μl reaction volume. Primer set RJH482/483 detected a 208 bp amplicon spanning the junction of exon 9 and 10 with a primer efficiency of 102%, measured at an annealing temperature of 64 °C and primer concentration of 100 nM each. Primer sequences were RJH482 5′-GCCACGTCCTACGTCATCAATCC and RJH483 5′-GGAATGCCGGGATGACCTGATAC. The primer set CW057/058 detected a 189 bp amplicon within exon 11 with a primer efficiency of 98.5% at melting temperature of 62 °C and primer concentrations of 200 nM each. Primer sequences were CW057 5′-GCCTGATGACCGATGTCTTTGG and CW058 5′-CGATTTCCTGCTGCTGCTCC. The RPL32 mRNA served as the internal reference and was detected using the primers RC133 5′-GCCCAAGGGTATCGACAACA and RC134 5′-GCGCTTGTTCGATCCGTAAC with an efficiency of 97% at 100 nM each and annealing temperature of 65 °C. The qPCRs were performed in a CFX96 instrument (Bio-Rad) using the following cycling parameters: step 1: 3 min at 98 °C, step 2: 10 s at 95 °C, step 3: 30 s at 62–65 °C, step 4: 40 s at 72 °C and fluorescence measurements taken, step 5: melt curve from 60 to 90 °C and image, with steps 2–4 repeated 39 times. The fold change in *Pumilio* mRNA was calculated using the measured Ct values according to the method established by Pfaffl (46).

For analysis of tethered function Nluc 2xMS2BS reporter mRNA in Fig. S1, total RNA was purified *via* Maxwell RSC simply RNA tissue kit, with slight alterations: (1) 1 ml aliquots of the cells, transfected with Nluc 2xMS2, Fluc, and indicated test protein, were harvested at 1000*g* for 3 min at room temperature and washed with 1 ml ice-cold 1× PBS prior to homogenization. (2) On-bead DNase I digestion used 10 μl of the provide DNase I solution. To remove residual plasmid DNA, 5 μg of the purified RNA was treated with Turbo DNase (Thermo) following the manufacturer’s instructions. Treated RNA was then purified using the RNA Clean and Concentrator kit (Zymo). LunaScript RT (NEB) was used to reverse transcribe 1 μg of DNase-treated RNA, using random hexamer primers, following the manufacturer’s instructions. As negative controls, equivalent reactions were performed in the absence of template cDNA or without the RT enzyme. To quantitate Nluc 2xMS2BS mRNA, 5 μl of cDNA was amplified using Luna Universal qPCR Master Mix (NEB) with 250 nM of oligonucleotide primers Nluc (forward: 5′-GTCCTGAGCGGTGAAAATGG) and Nluc (reverse: 5′-CGTAACCCCGTCGATTACCA). The following cycling parameters were used for amplification: 95 °C 2 min, (1) 95 °C 15 s, (2) 65 °C 1 min, (3) 60 °C 40 s, with 39 repeats of steps 1–3 and fluorescence measurements taken at step 3. This protocol produced a 94.3% amplification efficiency. To measure the internal control Fluc mRNA, 5 μl of cDNA was amplified with 250 nM of oligonucleotide primer Fluc (forward: 5′-GATCCTCAACGTGCAAAAGAAGC; reverse: 5′-TCACGAAGGTGTACATGCTTTGG). Fluc amplification used the cycling parameters: 95 °C 1 min, (1) 95 °C 15 s, (2) 63 °C 1 min, with 39 repeats of steps 1 to 3 and fluorescence measurements taken at step 2. This protocol produced a 93.7% amplification efficiency. The fold change in Nluc 2xMS2 mRNA, normalized to internal control Fluc, was calculated using the measured Ct values according to the Pfaffl method (46).

## Data availability

All data are contained within the article.

## Supporting information

This article contains supporting information (5, 8, 9).

## Conflict of interest

The authors declare that they have no conflicts of interest with the contents of this article.

## Supporting information

Table S1 **Data and statistics for Figures 2, 3, 4, 5, and 10**.

Table S2 **Plasmids**.

Figure S1 **Conserved phenylalanines F1033 and F1040 of Pumilio RD3 are necessary for reduction of reporter mRNA level in the tethered function assay**. Reduction in the level of the Nluc 2xMS2BS reporter mRNA by MS2 fusions of wild type or the F1033A+F1040A mutant RD3 was measured using RT-qPCR analysis, relative to the negative control MS2-EGFP. Nluc 2xMS2BS was normalized to the internal control Fluc in each sample. Mean and replicate log2 fold change values in mRNA levels, calculated using the ΔΔCt method, are graphed. N=3, ± StDev. For significance calling, p < 0.0001 = ∗∗∗∗ based on two-tailed unpaired Student’s *t* test. Western blot confirming the expression of each test protein is shown at the bottom. MS2 fusion proteins were detected via their C-terminal V5 epitope tag. Tubulin served as a loading control

Figure S2 Western blot analysis of bait (Panel **A**) and prey (Panel **B**) protein fusions expressed in yeast two hybrid strain, Y2H Gold. Bait proteins were expressed as fusions to the Gal4 DNA binding domain and had a Myc epitope tag. Prey proteins were expressed as fusions to the Gal4 transcription activation domain and had an HA epitope tag. Protein content and loading of the yeast cell extracts was assessed by staining the membranes with Sypro Ruby protein stain

Figure S3 **Verification of Not1-Flag and Pumilio knockout DL1 cell lines**. **A)** Sequence chromatogram of the Not1 gene with 3×Flag tag that was engineered onto the C-terminus of the Not1 coding region in DL1 cells. A TEV site was included between the Not1 and 3×Flag tag coding regions. **B)** Diagram of the *Pumilio* coding region depicting the location of the CRISPR-Cas9 targeted region and primer sets used for PCR, sequencing, and RT-qPCR detection. **C)** Exon 9 of the *Pumilio* (*Pum*) gene was PCR amplified in wild type DL1 and *Pumilio* KO clonal DL1 cell lines. The gel image was cropped to show the relevant lanes, as indicated by the dashed vertical line. **D)** The products in C were sequenced to confirm the wild type and *Pumilio* KO genotype, as shown by the aligned chromatograms. The position of the single guide RNA used for CRISPR-Cas9 genome engineering is shown at the top, along with the protospacer adjacent motif (PAM). The 10 base pair deletion in the *Pumilio* KO is highlighted. **E)** Diagram of the Pumilio protein produced *Pumilio* KO cell line. The 10 base pair deletion creates a frameshift after amino acid 725 (shown in red), resulting in a 765 amino acid non-functional protein product that lacks domains PCMb, RD3, and RBD. **F)** RT-qPCR analysis, using primer sets in exon 9 and exon 11 as shown in panel B, measured the reduction of Pum mRNA in the *Pumilio* KO relative to the wild type DL1 cells. Mean log2 fold change values are reported. N=3
