# Supplementary figures and images for: A conserved domain of Drosophila RNA-binding protein Pumilio interacts with multiple CCR4–NOT deadenylase complex subunits to repress target mRNAs
Source: J Biol Chem. 2022 Jul 16;298(9):102270. doi: 10.1016/j.jbc.2022.102270 (PMC9418443; doi:10.1016/j.jbc.2022.102270)

**Figure S1**  
Haugen et al.

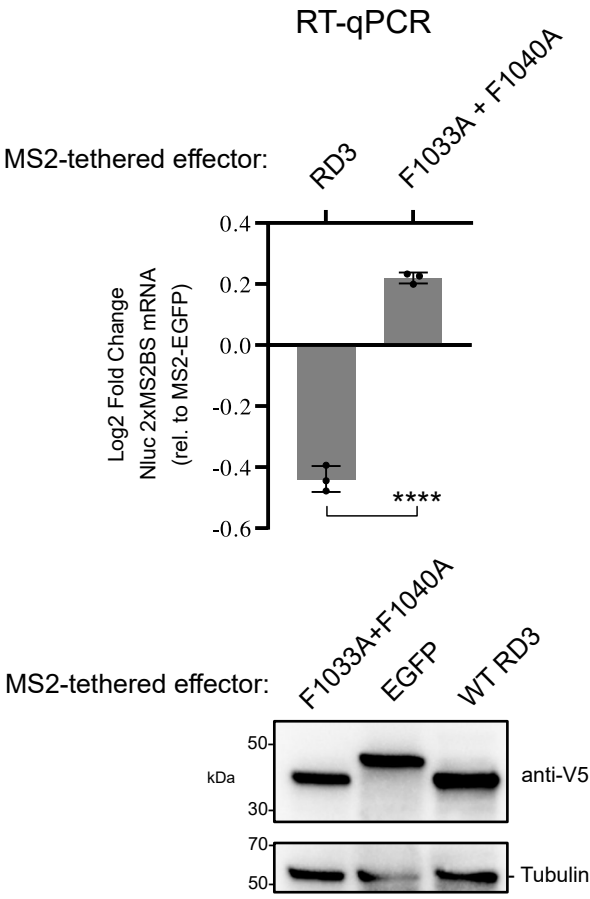

Supplement: Figure S1 — Conserved phenylalanines F1033 and F1040 of Pumilio RD3 are necessary for reduction of reporter mRNA level in the tethered function assay. Reduction in the level of the Nluc 2xMS2BS reporter mRNA by MS2 fusions of wild type or the F1033A+F1040A mutant RD3 was measured using RT-qPCR analysis, relative to the negative control MS2-EGFP. Nluc 2xMS2BS was normalized to the internal control Fluc in each sample. Mean and replicate log2 fold change values in mRNA levels, calculated using the ΔΔCt method, are graphed. N=3, ± StDev. For significance calling, p < 0.0001 = ∗∗∗∗ based on two-tailed unpaired Student’s t test. Western blot confirming the expression of each test protein is shown at the bottom. MS2 fusion proteins were detected via their C-terminal V5 epitope tag. Tubulin served as a loading control [file mmc3.pdf]

**Figure S2**  
Haugen et al.

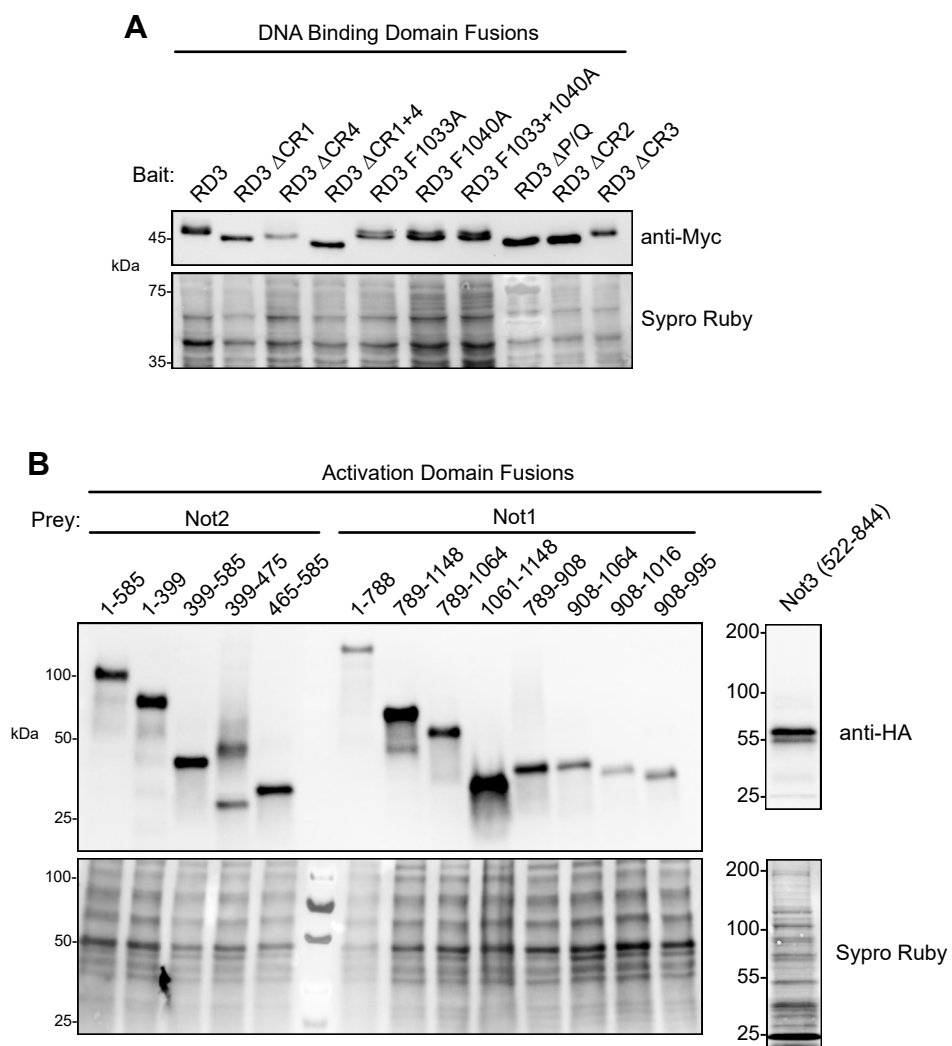

Supplement: Figure S2 — Western blot analysis of bait (Panel A) and prey (Panel B) protein fusions expressed in yeast two hybrid strain, Y2H Gold. Bait proteins were expressed as fusions to the Gal4 DNA binding domain and had a Myc epitope tag. Prey proteins were expressed as fusions to the Gal4 transcription activation domain and had an HA epitope tag. Protein content and loading of the yeast cell extracts was assessed by staining the membranes with Sypro Ruby protein stain [file mmc4.pdf]

Figure S3  
Haugen et al.

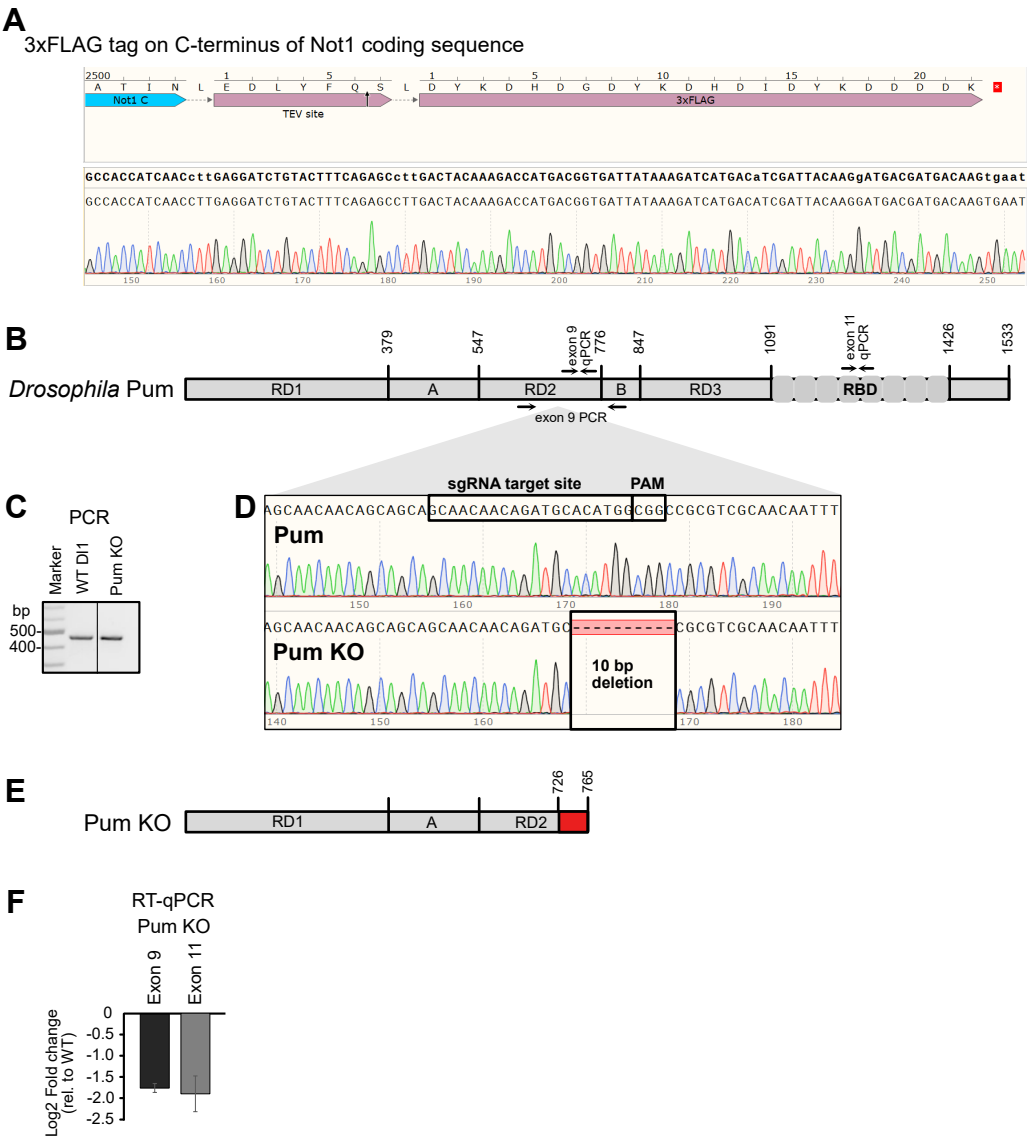

Supplement: Figure S3 — Verification of Not1-Flag and Pumilio knockout DL1 cell lines. A) Sequence chromatogram of the Not1 gene with 3×Flag tag that was engineered onto the C-terminus of the Not1 coding region in DL1 cells. A TEV site was included between the Not1 and 3×Flag tag coding regions. B) Diagram of the Pumilio coding region depicting the location of the CRISPR-Cas9 targeted region and primer sets used for PCR, sequencing, and RT-qPCR detection. C) Exon 9 of the Pumilio (Pum) gene was PCR amplified in wild type DL1 and Pumilio KO clonal DL1 cell lines. The gel image was cropped to show the relevant lanes, as indicated by the dashed vertical line. D) The products in C were sequenced to confirm the wild type and Pumilio KO genotype, as shown by the aligned chromatograms. The position of the single guide RNA used for CRISPR-Cas9 genome engineering is shown at the top, along with the protospacer adjacent motif (PAM). The 10 base pair deletion in the Pumilio KO is highlighted. E) Diagram of the Pumilio protein produced Pumilio KO cell line. The 10 base pair deletion creates a frameshift after amino acid 725 (shown in red), resulting in a 765 amino acid non-functional protein product that lacks domains PCMb, RD3, and RBD. F) RT-qPCR analysis, using primer sets in exon 9 and exon 11 as shown in panel B, measured the reduction of Pum mRNA in the Pumilio KO relative to the wild type DL1 cells. Mean log2 fold change values are reported. N=3 [file mmc5.pdf]
